# 134 Patients’ perspectives on determinants of physical activity promotion for chronic disease in primary care: A COM-B analysis

**DOI:** 10.1093/eurpub/ckae114.094

**Published:** 2024-09-26

**Authors:** Aisling McGrath, Barry Lambe, Evan Matthews, Karolyn McDonnell, Michael Harrison, Bróna Kehoe

**Affiliations:** Centre for Health Behaviour Research, Department of Sport and Exercise Science, South East Technological University, Waterford, Ireland; Centre for Health Behaviour Research, Department of Sport and Exercise Science, South East Technological University, Waterford, Ireland; Centre for Health Behaviour Research, Department of Sport and Exercise Science, South East Technological University, Waterford, Ireland; National Centre for Men’s Health, South East Technological University, Carlow, Ireland; Centre for Health Behaviour Research, Department of Sport and Exercise Science, South East Technological University, Waterford, Ireland; Centre for Health Behaviour Research, Department of Sport and Exercise Science, South East Technological University, Waterford, Ireland

## Abstract

**Purpose:**

Chronic disease (CD) accounts for more than half of the overall global disease burden and physical activity (PA) is an established evidence-based strategy for the prevention and management of CD. Global policy emphasises the value of embedding PA into primary healthcare, highlighting the positive effects on PA behaviour. Despite this, there is limited implementation of healthcare protocols for the management of PA in primary care, and further research is needed to guide its integration into routine practice. At an individual level, the voice of the patient living with CD is underrepresented in the literature, resulting in the absence of critical insights into determinants of PA promotion in primary care. The purpose of the research was to identify the patient perspectives on the determinants of PA promotion in primary care and to map these determinants across the six COM-B constructs.

**Methods:**

Semi-structured interviews (n = 22), guided by the COM-B model were conducted with people aged 35-60 years, at risk of or living with CD and not meeting the PA guidelines. A hybrid analytic approach of thematic inductive and deductive analysis was applied to the participant transcripts guided by a COM-B informed coding framework.

**Results:**

In total, 37 determinants across constructs related to capability, opportunity and motivation were prominent such as; physical capability constraints, the conflation of exercise with weight management, credibility of the health services in PA advice, communication styles in PA promotion, expectations of tailored support for PA, social support, accessibility, and integration of PA into routine habits.

**Conclusions:**

Exploring the determinants of PA promotion through the lens of the COM-B model facilitated a systematic approach to understanding the patient perspective of the healthcare professional broaching the topic of PA. Findings emphasise the value of HCPs being supported to broach the issue of PA in a therapeutic and patient-centred manner using diverse and flexible approaches, while highlighting the importance of tailored, accessible PA opportunities that build self-efficacy and foster social support. The research provides valuable learnings to support PA promotion and the development of strategies in primary care through encompassing the patient’s perspective.

**Funding Source:**

Health Service Executive Ireland.

